# Comparison of Laparoscopic and Laparotomic Total Hysterectomy in Terms of Patient Satisfaction and Cosmetic Outcomes

**DOI:** 10.3390/jcm14217646

**Published:** 2025-10-28

**Authors:** Suheyla Erbasaran Aydin, Turhan Aran, Suleyman Guven

**Affiliations:** 1 Department of Obstetrics and Gynecology, Vakfikebir State Hospital, 61100 Trabzon, Turkey; 2 Department of Obstetrics and Gynecology, Faculty of Medicine, Karadeniz Technical University, 61100 Trabzon, Turkey; turhanaran@ktu.edu.tr (T.A.); sguven@ktu.edu.tr (S.G.)

**Keywords:** cosmetic outcomes, laparoscopic hysterectomy, laparotomic hysterectomy, patient satisfaction, scar

## Abstract

**Background/Objectives**: Hysterectomy is the most common gynecologic surgical procedure. While extensive research has been conducted on the advantages of laparoscopy, the gynecology literature lacks sufficient studies on scar-related outcomes, patient satisfaction, and cosmetic outcomes. In this regard, this study aimed to compare cosmetic outcomes and patient satisfaction between laparotomy and laparoscopic hysterectomy cases performed at our tertiary university hospital center. **Methods**: Patients who underwent hysterectomy for benign gynecologic reasons were included in the study. The study group consisted of patients who had surgery via the laparoscopic technique, while the control group comprised patients who had laparotomy through a transverse abdominal incision (Pfannenstiel). Postoperative scar areas, scar thickness, color, height, and pain scores were evaluated after the 12th postoperative month. A digital caliper was used to calculate the scar area. Scar satisfaction and general body perceptions were assessed using questionnaires. **Results**: The mean scar area was significantly lower in the study group (*p* = 0.003). The physician’s scar assessments revealed no significant differences between the Manchester Scar Scale, POSAS Observer Scale, Vancouver Scar Scale, and SCAR Scale. The mean POSAS Patient Scale score, which assesses patients’ opinions of postoperative scars, was significantly lower in the study group than in the control group. In contrast, the Body Image Questionnaire score was higher (*p* < 0.01). There were no significant differences between the groups in mean Rosenberg Self-Esteem Scale and Body-Cathexis Scale scores. **Conclusions**: The patients in the study group were more satisfied with their scars but less satisfied with their body image. Contrary to general expectations, the patients were found to be less satisfied with the visible scar outcomes on the abdominal wall resulting from multi-port surgical procedures. Studies are needed to inform patients about scars before operations, select ports for use during operations, and evaluate the effect of the port-site surgical repair technique on cosmetic outcomes.

## 1. Introduction

Hysterectomy is the most common gynecologic surgical procedure. Most of the indications are benign causes such as endometriosis, uterine prolapse, abnormal uterine bleeding, and myoma uteri [1]. Hysterectomy methods can be divided into two groups: laparotomy and minimally invasive surgery. Minimally invasive surgical procedures are classified into vaginal route, laparoscopy, laparoscopically assisted vaginal hysterectomy, and robotically assisted laparoscopy. After the indication for hysterectomy is established, the surgical method to be used is determined based on the patient’s clinical condition, the expected benefit, the surgeon’s professional experience, the patient’s anatomical suitability for the method, and the equipment and resources available at the surgical center. Total laparoscopic hysterectomies (TLHs) are more commonly performed than vaginal hysterectomies (VHs) due to their easier access to the adnexa and increased surgical visibility [2,3]. The literature suggests that laparoscopic hysterectomies or laparoscopically assisted vaginal hysterectomies, compared to abdominal hysterectomies, significantly reduce morbidity, blood loss, and analgesic requirements, shorten hospitalization, provide better cosmetic outcomes and faster recovery, and considerably decrease scar infections [4] Although laparoscopic surgery has disadvantages such as longer operative time, higher cost, and increased risk of ureteral injury [5,6], gynecologists more widely adopt it due to better cosmetic outcomes and shorter hospitalization duration. It is preferred in patients without extensive adhesions and significant adnexal pathology. Other reasons for the greater preference for laparoscopic surgery include low postoperative pain scores and earlier return to daily activities for patients [3].

Today, minimally invasive approaches play a significant role in the surgical treatment of benign and malignant gynecological conditions. In this context, they are preferred and performed more frequently than conventional open surgery. The most commonly used minimally invasive approaches include laparoscopic hysterectomy, robotic-assisted hysterectomy, and laparoscopic-assisted vaginal hysterectomy. These techniques offer many advantages over open surgery, including reduced perioperative morbidity, shorter hospital stay, faster recovery and return to work, and lower complication rates. In cancer cases with significant systemic effects, minimally invasive surgical approaches offer substantial benefits compared to open surgery in terms of recovery time and complication rates. However, definitive results regarding oncological outcomes are not available [7]. Although minimally invasive surgery is known to be advantageous from a clinician’s perspective and objective data, the patient’s perspective on these procedures and postoperative scar formation is unclear. It is unclear how the patient internally evaluates the surgical procedures. This is what our research was designed to address.

Minimally invasive hysterectomy techniques are also evolving. Hysterectomy, previously performed with four or five abdominal ports, is now performed with a single port opened through the umbilicus. Another recent study compared outcomes of single-port robotic hysterectomy with those of single-port laparoscopic hysterectomy. Colpotomy time was shorter with robotic hysterectomy, whereas vaginal cuff closure time was longer. When comparing objective outcome parameters, both techniques yield similar results. Both approaches also yield similar operative times. Minimally invasive methods remain preferred, particularly for benign gynecological conditions [8].

While extensive research has been conducted on the surgical outcomes of laparoscopy (blood loss, complications, length of stay, etc.), the gynecology literature lacks sufficient studies on scar area, patient satisfaction, and cosmetic outcomes. In this regard, this study aimed to compare cosmetic outcomes and patient satisfaction between laparotomy and laparoscopic hysterectomy performed at our tertiary university hospital center.

## 2. Materials and Methods

### 2.1. Participants

This study was conducted at a tertiary hospital among patients who underwent hysterectomy for benign gynecologic conditions and volunteered to participate. The study commenced after obtaining approval from the Karadeniz Technical University Clinical Research Ethics Committee (2020/65). Patients who underwent hysterectomy via laparoscopic technique were included in the study group, while those undergoing hysterectomy through a lower abdominal transverse (Pfannenstiel) incision were assigned to the control group. All surgeries were performed by the same surgical team using the same techniques, number of ports, and port sizes. The primary closure of the port sites was also performed using the same method. For laparoscopic hysterectomy operations, a 10 mm trocar was inserted through the umbilicus for optical placement. Four auxiliary trocars, each 5 mm in size, were placed, with two positioned 2 cm lateral and 2 cm superior to the anterior superior iliac spine, and two located approximately 8 cm away from these positions in the periumbilical area (5-port technique). All patients were operated on under general anesthesia.

### 2.2. Inclusion–Exclusion Criteria

The exclusion criteria were a history of laparotomy, diabetes mellitus or hypertension that could affect wound healing, endocrinopathy, connective tissue disease, gynecologic or non-gynecologic malignancy detection during the postoperative period, intraoperative bladder or bowel rupture, or organ/vascular injury, postoperative wound infection, pelvic abscess, or febrile complications, a body mass index (BMI) > 35 kg/m^2^, the requirement of reoperation for reasons unrelated to the initial surgery, and psychiatric or mood disorders.

**Figure 1:**
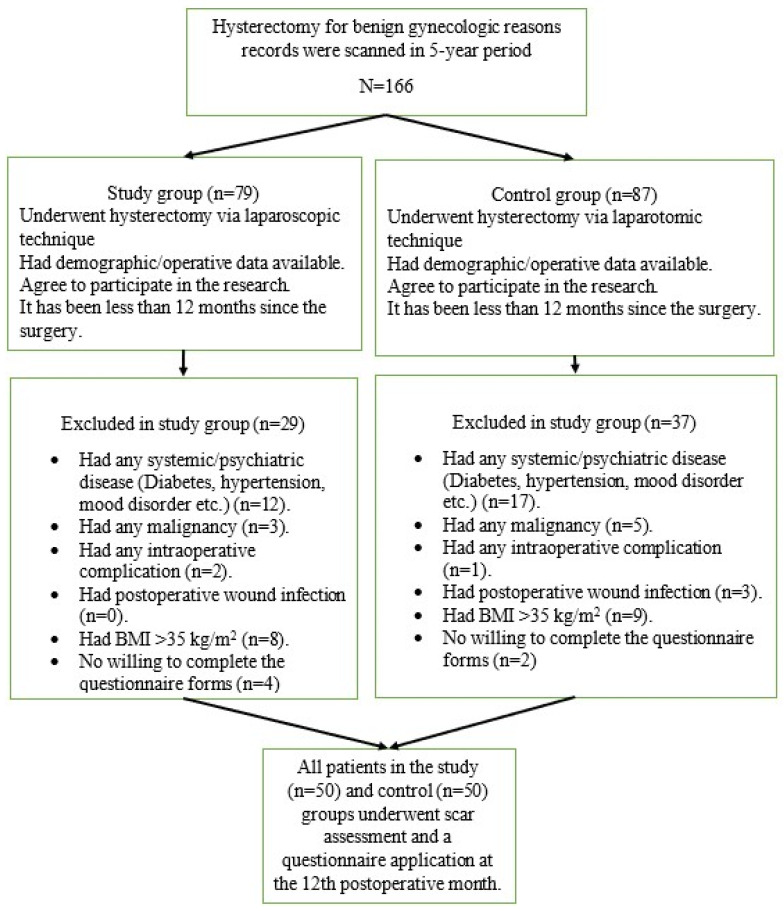
Patient selection flowchart.

Cases that met the inclusion and exclusion criteria for the study were evaluated over 5 years. The patient selection flow chart is shown in Figure 1. Case recruitment was terminated once 50 cases were recruited for each group. All cases meeting the inclusion criteria were recalled for evaluation and survey administration at 12 months postoperatively.

### 2.3. Investigation Procedures

The patients’ age, weight, height, BMI, gravidity, parity, postoperative follow-up duration, uterine volume, uterine weight, hospitalization duration, antibiotic use duration, operation duration, and amount of blood loss were recorded. Scar and patient satisfaction assessments were made after the 12th postoperative month. Scar lengths were measured by a single physician (SEA) using a digital caliper. Scar areas were calculated. Scar color, height, and tenderness were assessed. In addition to scar area measurement, scar assessments were performed using the Vancouver Scar Scale [9], Manchester Scar Scale [10], POSAS (Patient and Observer Scar Assessment Scale) [11], and Scar Cosmesis Assessment and Rating Scale [12]. The Rosenberg Self-Esteem Scale [13], the Body-Cathexis Scale [14], and the Body Image Questionnaire [15] were used to assess patient satisfaction and general perceptions.

### 2.4. Manchester Scar Scale

The Manchester Scar Scale, published by Beausang et al. in 1998 [10], introduced two new items: (1) an “overall assessment”, rated on a VAS scale (0–10), and (2) whether the scar appears matte or shiny. The Manchester Scar Scale consists of parameters such as color, contour, distortion, and scar texture [10].

Patients assign scores ranging from 1 to 4 for these parameters. They also assign scores from 1 to 2 depending on whether the scar is shiny or matte. On the 10 cm horizontal line at the top of the test, they mark between the poles representing an excellent wound and a poor wound. Each patient receives a score between 5 and 28 points. A lower score indicates a better scar, while a higher score indicates a poorer scar (Table S1, available as a Supplementary File).

### 2.5. Patient and Observer Scar Assessment Scale (POSAS Patient)

In 2004, the Patient and Observer Scar Assessment Scale (POSAS) was developed, incorporating patient opinion on visual and physical scar characteristics, as well as symptoms such as pain and itching. POSAS has proven helpful in demonstrating both the observer’s and the patient’s perspectives on multiple characteristic scar quality [11].

In the POSAS Patient Scale, the patient rates, on a scale of 1 to 10, whether the scar is itchy or painful a few weeks after the operation. One indicates “no pain at all”, while 10 indicates “very much”. Also, the patient indicates the color, height, thickness, and image of the skin by assigning a score from 1 to 10 (Table S2, available as a Supplementary File).

### 2.6. Patient and Observer Scar Assessment Scale (POSAS Observer)

In the POSAS Observer Scale, the observer assigns the patient a score from 1 to 10 for six parameters. The patient’s score ranges from 6 to 60 points. While a score of 6 reflects normal skin, a score of 60 reflects the worst scar imaginable [11]. The observer assesses the patient’s scar for vascularity, pigmentation, thickness, height, and pliability (Table S3, available as a Supplementary File).

### 2.7. Vancouver Scar Scale

In the late 1980s, the first concepts of scar assessment scales were introduced. In 1990, the Vancouver Scar Scale, the first widely used and validated scar scale, was developed by Sullivan et al. [9]. The scale is based on the observer’s assessment of four parameters—this test scores vascularity, pigmentation, pliability, and height. Patients receive a score between 0 and 13, with 0 indicating a scar characteristic of a regular skin pattern and 13 reflecting a more complex scar (Table S4, available as a Supplementary File).

### 2.8. Scar Cosmesis Assessment and Rating (SCAR) Scale

The Scar Cosmesis Assessment and Rating (SCAR) scale, with six observer components and two patient components, was developed by J. Kantor in a 2016 study. While observer and patient assessments can be reported separately in POSAS, the SCAR scale overall score is calculated by combining the observer and patient sections [12] (Table S5 provided as a Supplementary File).

### 2.9. Body Image Questionnaire

Only the five questions were used for the published body image scale [14]. It is a questionnaire assessing postoperative patient satisfaction and perceptions, comprising five questions, each rated on a 4-point scale (Table S6 provided as a Supplementary File).

### 2.10. Body-Cathexis Scale

The study used the Turkish version of the Body-Cathexis Scale (BCS) to assess patients’ perceptions of their bodies [15]. The scale provides an objective measure of people’s feelings towards various aspects of their bodies. It assesses their satisfaction with 40 different body parts or functions (e.g., arm, leg, face, or a specific function of the face, such as sexual activity level).

The original scale consists of 46 items, but its Turkish version uses a five-point Likert-type scale with 40 items (5 = I strongly like it, 4 = I moderately like it, 3 = I’m neutral, 2 = I don’t like it very much, 1 = I don’t like it at all) (Table S7 in the Supplementary File). The most positive statement is assigned 5 (five) points, while the most negative statement is assigned 1 (one) point. With this assessment, the lowest possible total score is 40, while the highest possible total score is 200. A rise in the total score indicates increased satisfaction with body parts or functions, while a fall in the total score indicates decreased satisfaction.

### 2.11. Rosenberg Self-Esteem Scale

The Rosenberg Self-Esteem Scale has been used since 1965. It is frequently used in social sciences research. It was published in Morris Rosenberg’s book titled Society and the Adolescent Self-Image. This 10-item scale is the most widely used measure of self-esteem (Table S8 in the Supplementary File). The measure was graded on a four-point scale, and mean scores were calculated, with higher scores indicating higher self-esteem [13].

### 2.12. Statistical Analysis

Descriptive statistics of the assessment results are presented in numbers and percentages for categorical variables and as means and standard deviations for numeric variables. The data’s compatibility with normal distribution assumptions was evaluated using the Kolmogorov–Smirnov test. In comparisons of numeric variables between two independent groups, Student’s *t*-test was used for normally distributed data. Differences between the rates of categorical variables in independent groups were analyzed using the chi-square test. The statistical significance level was set at *p* < 0.05 for all hypothesis tests. *p*-values were based on two-tailed tests for the *t*-test and chi-square analyses. Statistical analyses were performed using SPSS 11.5 software. The sample size and power analyses were conducted using G*Power 3.1.9.7. Based on our preliminary study results, the effect size was set at 0.668, with at least 50 cases per group, a power of 0.95, and an alpha error rate of 0.05.

## 3. Results

A total of 100 patients were assessed. The study group consisted of 50 patients who underwent laparoscopic total hysterectomy, while the control group comprised 50 patients having total abdominal hysterectomy. Demographic data and clinical characteristics of the patients are given in Table 1. All cases in the study and control groups were of asian and white races. There were no significant differences between the groups in mean weight, height, BMI, gravidity, parity, time to postoperative scar assessment, uterine volume, uterine weight, or postoperative antibiotic use. While age was found to be statistically significantly higher in the laparoscopic hysterectomy group compared to the laparotomy hysterectomy group, the duration of hospital stay and uterine size were found to be statistically significantly higher in the laparotomy group. Compared with the control group, the study group had a longer surgical duration, while blood loss was lower.

The total mean scar area was significantly lower in the study group (laparoscopic hysterectomy) compared to the control group (total abdominal hysterectomy) (*p* = 0.003). However, no significant differences were found between the groups in terms of scores on the Manchester Scar Scale, POSAS Observer Scale, Vancouver Scar Scale, and SCAR Scale, as assessed by the physician (Table 2).

The mean POSAS Patient Scale score, which assesses patients’ opinions of postoperative scars, was significantly lower in the study group than in the control group. In comparison, the Body Image Questionnaire (BIQ) score was higher (19.16 ± 1.87 vs. 17.68 ± 2.61, 95% CI (0.578–2.38), *p* < 0.01). Higher POSAS and BIQ scores indicated worse patient opinion regarding the scare and their overall image, respectively. There were no significant differences between the groups in mean Rosenberg Self-Esteem Scale and Body-Cathexis Scale scores (Table 3).

## 4. Discussion

Laparoscopy is increasingly being offered to patients as a treatment option due to its many advantages and good cosmetic outcomes. Although many studies have shown vaginal hysterectomy to be superior to all other methods in terms of cosmetic postoperative parameters, laparotomic hysterectomy is still the most preferred method. On the other hand, laparoscopic surgeries continue to gain popularity and become more common. In our study, we found negative survey responses from patients regarding the cosmetic and satisfaction aspects of minimally invasive surgical techniques, contrary to physicians’ perceptions. We concluded that while the patients were more satisfied with their scars and various parameters, such as postoperative pain, hospitalization duration, and rapid return to daily life, compared to laparoscopic cases, they were less confident in their body image.

In our study, we aimed to compare laparoscopy, a minimally invasive surgical technique we recommend for its many advantages, with abdominal hysterectomy cases in terms of cosmetic outcomes and patient satisfaction, and to evaluate patients’ perceptions of these commonly used techniques. Studies on reducing the number of ports or port size in laparoscopic operations for non-gynecologic abdominal operations have reported better cosmetic outcomes and improved patient satisfaction. There have been few studies in the literature comparing laparoscopy cases for gynecologic indications with other surgical methods regarding cosmetic outcomes and patient satisfaction. Therefore, our study was unique and contributed to the literature with its results.

All cases in this study were selected from those requiring hysterectomy for benign reasons. As is well known, cancer cases present with systemic effects, a chronic course, prolonged surgery times, the need for extensive surgical techniques, and the presence of multiple coexisting systemic diseases. These factors can also introduce bias and inaccurate assessments of wound healing [16]. For all these reasons, our study group consisted solely of benign cases.

The choice of surgical technique is critical in overweight cancer patients, considering perioperative and postoperative complications. In cervical cancer patients undergoing radical hysterectomy, laparoscopic surgery offers significant advantages over open surgery. Less intraoperative blood loss, shorter hospital stays, and less fatty tissue liquefaction have been reported with laparoscopic hysterectomy. Even in such challenging cases, the laparoscopic approach yields promising results [17]. This study showed that the laparoscopic approach has a clear advantage in both benign and complex malignant cases.

The Pfannenstiel incision is the preferred skin incision for patients undergoing abdominal hysterectomy for benign indications. It is a transverse skin incision about 2 cm above the symphysis pubis, extending towards the anterior superior iliac spine and located 2–3 cm medial to it. In many studies, the Pfannenstiel incision is not superior to other abdominal incisions in terms of postoperative pain, early intestinal peristalsis, early ambulation without assistance, reduced analgesic requirements, or contribution to abdominal wall strength [18,19]. Nevertheless, the Pfannenstiel incision is often preferred because it leads to better cosmetic outcomes. In one study, the cosmetic outcomes of surgery performed through the Pfannenstiel incision were compared with those of midline and laparoscopic methods. They observed better wound healing and cosmetic outcomes with the Pfannenstiel incision [20].

Laparoscopic hysterectomy can be performed through a single incision or via conventional multiport laparoscopy. Multiport laparoscopy is a procedure involving the insertion of a 10 mm trocar through the umbilicus as well as a total of four 5 mm trocars, with two placed bilaterally in each lower quadrant. Good cosmetic outcomes are also achieved with subcuticular skin closure. The single-incision laparoscopic surgical technique is referred to as embryonic natural orifice transumbilical endoscopic surgery [21], with the incision concealed within the umbilicus and limited to a single cut, resulting in an almost scarless outcome. In comparing these two techniques, one author reported better cosmetic outcomes with a single-incision total hysterectomy. The authors reported better cosmetic outcomes with safer outcomes compared to conventional multiport surgery [21].

In recent years, scar assessment scales have been developed to assess the effectiveness of treatments and their impact on patients. Clinician-administered scales assess observable aspects of scars, while patient-administered scar scales assess additional aspects that clinicians cannot observe, such as scar symptoms and quality of life. Unlike measurement devices, scar assessment scales provide qualitative assessments of multiple scar characteristics by an individual, whether a patient or a clinician. Scar assessment scales are sometimes criticized for their subjective nature. However, they typically have advantages such as ease of accessibility, the ability to capture the patient’s views on their scars, and enabling rapid assessment of multiple scar characteristics. They are also crucial because patients today are more informed about treatment options, more involved in making treatment decisions, and more active in reporting treatment outcomes.

In clinical practice, patients with high cosmetic concerns often prefer a laparoscopic operation. Abdominal hysterectomy patients who do not have problems regarding scar formation usually do not experience dissatisfaction due to their surgical incisions being located in the bikini area. In TLH cases, the presence of scars in five distinct visible regions of the anterior abdominal wall may decrease satisfaction levels. A study involving 132 patients undergoing mini-laparoscopic or conventional laparoscopic surgery examined operative time, mean blood loss, preoperative and postoperative complications, POSAS (Patient and Observer Scale) scores, and hospitalization duration. The study found no statistically significant difference between the two groups in preoperative and postoperative complications or hospitalization duration [22]. While there were substantial differences in operative time and hematocrit change, both POSAS Patient and POSAS Observer scar scores were higher in the mini-laparoscopy group (*p* < 0.05).

Conducting a meta-analysis in 2020, one author evaluated patient satisfaction, chronic pain, and cosmetic outcomes in single-port laparoscopic and multi-port laparoscopic surgical cases over the long term. They found that although many studies detected statistically significant differences in POSAS Patient Scale scores in the short term, these differences did not persist in the long term (*p* = 0.852) [23]. It was reported that reducing the number and size of ports was effective in improving pain scores during laparoscopic surgery [24,25]. In a multicenter randomized controlled trial, postoperative morbidity and cosmetic outcomes were compared between multi-port and single-port laparoscopy. Compared to the patients, the surgeons found scar shape and skin retraction more aesthetically acceptable [26]. In our study, we found that the POSAS Patient scar score was significantly lower in laparoscopic hysterectomies than in abdominal hysterectomies. Although the POSAS Observer scar score was lower in the laparoscopic surgery group, this difference was not statistically significant [23].

In one study, when body image scores in surgical cases with fewer ports were considered, patients were found to be more satisfied with their bodies, believing the operation caused less damage [27]. In our study, no significant differences were found between the two groups in mean Rosenberg Self-Esteem Scale and Body-Cathexis Scale scores. Although the patients were satisfied with their surgical scars, the Body Image Questionnaire score was significantly higher in patients who underwent laparoscopy. This score, which means less patient satisfaction, also reflects the perception that the operation damaged their body. They also reported feeling less attractive and less feminine compared to the laparotomy group. We think this is related to the established perception of scarless surgery among patients/society before laparoscopy. Additionally, in our clinic, primary skin closure is used as the skin-suturing method for laparoscopic hysterectomy cases. In many patients, scar areas intersecting at 90 degrees with the incision lines were observed in suture lines perpendicular to Langer’s lines. There were patients with suture-related scar formation greater than the surgical incision. This may have contributed to the patients’ poor body image in the study group.

It was also reported that reduced port size had a statistically significant effect on scar area, leading to smaller scars (*p* < 0.001) [25]. In a 2014 retrospective study, Kumar et al. [28] compared mini-laparotomy versus laparoscopy in gynecologic cases. They argued that mini-laparotomy has similar advantages to minimally invasive approaches. They found no statistical difference in total incision length (mm) between mini-laparotomy cases. In our study, the total scar area was significantly lower in the study group. In a retrospective study [29], similar to many others, laparoscopic surgery was compared with laparotomic surgery in patients with adnexal masses, and hospitalization duration was significantly shorter in laparoscopic cases. In our study, hospitalization duration was considerably shorter in laparoscopic cases (*p* < 0.001).

In one retrospective study comparing endometrial cancer cases subjected to laparoscopic and laparotomic techniques, Baum et al. highlighted the superiority of laparoscopy over open surgery in terms of overall morbidity, intraoperative complications, blood loss, postoperative recovery, and the incidence and severity of postoperative complications [30]. Postoperative hospitalization was significantly shorter in laparoscopic cases. In our study, uterine weight and volume were similar in both laparoscopic and laparotomic surgery groups. In our study, cases with at least one year elapsed since surgery were included to assess long-term outcomes. There was no significant difference in postoperative time between the two groups.

In a prospective study of gynecologic oncologic cases, postoperative intravenous antibiotic use was 13% in laparotomy patients and 8% in laparoscopy patients, with the laparotomy group showing a statistically significantly higher rate. Oral antibiotic use was reported as 16% and 12% in the laparotomy and laparoscopy groups, respectively, with the laparotomy group exhibiting statistically significantly higher use [31]. In our study, all patients received antibiotics postoperatively. There was no difference in the duration of antibiotic use between the groups.

In one study [32], laparoscopic and open surgery were compared for the development of intussusception in adults. Early oral intake and complication rates were found to be lower in laparoscopic surgeries compared to laparotomies. In terms of patients’ demographic data, those with hypertension were statistically significantly more prevalent in the laparoscopy group. In contrast, patients with diabetes mellitus were more common in the open surgery group, though the difference was not significant. In our study, to avoid bias, patients with chronic diseases such as diabetes mellitus that could affect wound healing were excluded.

In their study comparing mini-laparotomy and laparoscopy, Kumar A et al. [28] reported scar complications in 5 patients in the mini-laparotomy group and one patient in the laparoscopy group. Walker JL et al. [31], comparing laparoscopy and laparotomy in a gynecologic oncology patient group, found no statistically significant difference in scar infection rates.

In a Cochrane review of multicenter randomized controlled trials comparing laparoscopy and laparotomy for benign ovarian tumors, fewer postoperative complications and surgical side effects, such as fever and infection, were reported with laparoscopy [32]. In our study, we found postoperative fever in 7 laparotomic patients and two laparoscopic patients, with no statistical difference between the groups (*p* = 0.160).

Many studies have shown that products such as silicone sheets or gels, used to prevent hypertrophic wound formation, contribute to healing by increasing hydration in the skin layers. Additionally, intralesional applications of steroids or 5-fluorouracil (5-FU) were found to have beneficial results in preventing hypertrophic wounds or keloids [33]. In our study, topical cream use for postoperative scars was more common in the laparotomic group, but the difference was not statistically significant. Jina et al. [34] investigated the effectiveness of keratin gel in improving poor scars after median sternotomy. The Manchester Scar Scale (*p* = 0.025), POSAS Patient Scale (*p* = 0.01), and POSAS Observer Scale (*p* < 0.01) scores were statistically significantly higher in the group with poor wound healing. In contrast, no significant difference was found in the general group. When all scales were considered, there was no significant difference in scar assessment for the three groups. The authors attributed this to the lack of impact capacity for wounds that already exhibited good cosmetic outcomes and to the minimal room for improvement, as the wounds had low scores [35]. In our study, no statistically significant differences were found in the Manchester Scar Scale, POSAS Patient Scale, and POSAS Observer Scale scores.

The Vancouver Scar Scale is an old scar scoring test widely used as the Burn Scar Index in clinical practice and research to document the change in scar appearance [35]. Today, POSAS and the Manchester Scar Scale are more commonly used for linear scars. In a study evaluating the reliability and validity of the Vancouver Scar Scale, poor reliability was observed [36]. In our study, there was no difference in the Vancouver Scar Scale scores.

As with other gynecological surgeries, simpler, less traumatic, but more expensive surgeries produce better satisfaction scores [37]. However, laparoscopy may have disadvantages, including more pronounced complications. For example, while laparoscopic lymphadenectomy provides a more effective lymph node dissection, lymphatic fluid leakage may occur. Postoperative oil administration can be used to identify chylous tubes and prevent chylous leakage [38]. It has been reported that performing laparoscopic lymphadenectomy with the new extraperitoneal technique reduces complications [39]. Similarly, the differences in body image scores among the patients in our study suggest that the laparoscopy technique should be reevaluated.

The difference in Body Image Questionnaire scores between groups appears statistically significant. Moreover, the absolute difference between groups is slight, raising concerns about the clinical significance of this result, especially given that the study’s main conclusion relies on it. In contrast, all other patient-reported measures (Rosenberg Self-Esteem Scale, Body-Cathexis Scale, POSAS Patient Scale) show results that are comparable or more favorable for the laparoscopic group. This may be another limitation of the study. Future studies with larger series may support our results. The main reason for this situation may be that the subjects may have placed significant emphasis on the uterine organ in their perspective of sexual ability, and losing it may have caused them to feel considerable loss in body image. Also, the fact that such a valuable and essential organ was removed through tiny holes rather than a large incision may have led patients to feel this way about their body perception.

Future planned studies could focus on why laparoscopy, during a minor, comfortable surgery, has such negative consequences for body image. Specifically, qualitative interviews or mixed-methods studies could help elucidate why patients in the laparoscopic group reported poorer body image despite objectively better scar outcomes. Likewise, perhaps a more comprehensive psychological evaluation in further studies could answer this question.

It is plausible that preoperative perceptions or expectations regarding “scarless” surgery may have influenced postoperative self-image, or that patients who opted for the laparoscopic approach already had greater baseline body-image concerns. This represents a significant limitation and potential confounding factor. Because the subjects were selected by application order, the two groups had different mean ages. This was a limiting factor in the study. Perhaps the lack of significant differences between the two groups is explained by the relatively advanced age of the participants. While all patients may be self-conscious regarding body image, younger women may place significantly higher value on aesthetics, and thus a study with younger participants may reveal differences in scores. Another limitation of our study was the inclusion of patients who had undergone a hysterectomy at least 1 year prior. Despite a higher number of patients undergoing laparoscopic hysterectomy over 5 years, the COVID-19 pandemic led many patients to miss follow-up appointments or decline to participate in the study. Therefore, the number of patients remained limited.

## 5. Conclusions

Laparoscopic hysterectomy is increasingly recommended and performed for its many advantages. One of the main reasons for its preference is the belief in better cosmetic outcomes. Although studies on short-term outcomes of surgical scars suggest better scar cosmetics, others show the opposite in the long term. The patients in the study group were more satisfied with their scars but less satisfied with their body image. Contrary to our expectations, the patients were found to be less satisfied with the visible scar outcomes on the abdominal wall resulting from multiport surgical procedures.

## Figures and Tables

**Table 1:** Demographic data and characteristics in laparotomic and laparoscopic total hysterectomy groups.

	Control Group (LH)	Study Group (TLH)	*p* Value
Age (years)	49.34 ± 6.87	52.78 ± 7.66	**0.020**
Weight (kg)	78.80 ± 12.08	77.66 ± 15.51	0.683
Height (cm)	159.54 ± 5.23	159.98 ± 6.88	0.720
BMI (kg/m^2^)	31.01 ± 4.95	30.3783 ± 5.9583	0.568
Gravidity	3.82 ± 2.11	4.02 ± 2.57	0.672
Parity	3.12 ± 1.74	3.34 ± 2.08	0.568
Uterine volume (measurements during pathological examination)	970.12 ± 1051.32	644.74 ± 478.25	0.05
Uterus weight (grams)	273.14 ± 329.01	194.46 ± 140.71	0.125
Hospitalization duration (postoperative hospitalization duration)	3.84 ± 0.76	3.00 ± 1.09	**<0.001**
Duration of antibiotics use (days)	7.8 ± 1.47	7.62 ± 1.99	0.334
Operative time/Anesthesia time (min)	108.8 ± 21.5	164.0 ± 26.3	**<0.001**
Estimated blood flow (mL)	477.4 ± 52.3	152.0 ± 33.3	**<0.001**

LH: laparotomic hysterectomy, TLH: total laparoscopic hysterectomy; mean ± standard deviation is given for comparison. Student’s *t*-test was used for comparison. ± Referred standard deviation. The bold values referred sthe statistical significance.

**Table 2:** Comparison of total scar area and scar texture as assessed by the physician.

	Control Group (LH, n = 50)	Study Group (TLH, n = 50)	*p* Value
**Total scar area (mm^2^)**	157.51 ± 96.37	109.68 ± 57.01	**0.003**
**Manchester Scale Score**	9.56 ± 2.61	9.02 ± 3.08	0.347
**POSAS Observer Scale Score**	16.28 ± 5.77	14.80 ± 8.97	0.329
**Vancouver Scale Score**	4.40 ± 2.59	3.48 ± 2.67	0.084
**SCAR Scale Score**	5.88 ± 2.68	5.3 ± 3.18	0.327

LH: laparotomic hysterectomy, TLH: total laparoscopic hysterectomy. Mean ± standard deviation is given for comparison. Student’s *t*-test was used for comparison. ± Referred standard deviation. The bold values referred sthe statistical significance.

**Table 3:** Postoperative scar and body image assessment from patients’ perspective.

	Control Group (LH, n = 50)	Study Group (TLH, n = 50)	*p* Value
**POSAS Patient Scale Score**	15.78 ± 10.62	8.98 ± 3.53	**<0.001**
**Rosenberg Self-Esteem Scale Score**	30.8 ± 5.64	29.41 ± 5.19	0.200
**Body Image Questionnaire Score**	17.68 ± 2.61	19.16 ± 1.87	**0.002**
**Body-Cathexis Scale Score**	147.14 ± 21.80	149.48 ± 14.21	0.526

LH: laparotomic hysterectomy, TLH: total laparoscopic hysterectomy. Mean ± standard deviation is given for comparison. Student’s *t*-test was used for comparison. ± Referred standard deviation. The bold values referred sthe statistical significance.

## Data Availability

All data are available upon request from the corresponding author.

## Supplementary Materials

The following supporting information can be downloaded at: https://www.mdpi.com/article/10.3390/jcm14217646/s1, Table S1. Manchester Scar Scale [10]. Table S2. Patient Scar Assessment Scale (POSAS Patient) [11]. Table S3. Observer Scar Assessment Scale (POSAS Observer) [11]. Table S4. Vancouver Scar Scale [9]. Table S5. Scar Cosmesis Assessment and Rating Scale [12]. Table S6. Body Image Questionnaire [14]. Table S7. Body-Cathexis Scale [15]. Table S8. Rosenberg Self-Esteem Scale [13].

## Abbreviations

The following abbreviations are used in this manuscript:

TLH	Total laparoscopic hysterectomy
VH	Vaginal hysterectomy
LH	Laparotomic hysterectomy
BMI	Body mass index
POSAS	Patient and Observer Scar Assessment Scale
SCAR	Scar Cosmesis Assessment and Rating
BCS	Body-Cathexis Scale
