# Supplementary material for: Comparison of Laparoscopic and Laparotomic Total Hysterectomy in Terms of Patient Satisfaction and Cosmetic Outcomes
Source: J Clin Med. 2025 Oct 28;14(21):7646. doi: 10.3390/jcm14217646 (PMC12608123; doi:10.3390/jcm14217646)
Supplement: Supplementary file 1 [file jcm-14-07646-s001.zip › jcm-3925151-supplementary.pdf]

Journal of Clinical Medicine

Table S1. Manchester Scar Scale [10].

Visual Analog Scale			
Excellent			Poor
Lighter or darker (cf. to surrounding skin)	A	Excellent	1
		Slight mismatch	2
		Obvious mismatch	3
		Gross mismatch	4
Matte vs. shiny	B	Matte (1) / Shiny (2)	
Contour	C	Flush with surrounding skin	1
		Slightly proud/indented	2
		Hypertrophic	3
		Keloid	4
Distortion	D	None	1
		Mild	2
		Moderate	3
		Severe	4
Texture	E	Normal	1
		Just palpable	2
		Firm	3
		Hard	4

Table S2. Patient Scar Assessment Scale (POSAS Patient) [11].

**Patient scar assessment scale**											
**No, no complaints**	1	2	3	4	5	6	7	8	9	10	**Yes, worst imaginable**
Is the scar painful?											
Is the scar itching?											
**No, as normal skin**	1	2	3	4	5	6	7	8	9	10	**Yes, very different**
Is the color of the scar different?											
Is the scar stiffer?											
Is the thickness of the scar different?											
Is the scar irregular?											
Total score	Patient scar scale:										

Table S3. Observer Scar Assessment Scale (POSAS Observer) [11].

**Observer scar assessment scale**											
**Normal skin**	1	2	3	4	5	6	7	8	9	10	**Worst imaginable**
Vascularity											
Pigmentation											Hypo<br>Mixed

		Hyper
Thickness		
Height		
Pliability		
Total score	Observer scar scale	

Table S4. Vancouver Scar Scale [9].

PIGMENTATION				VASCULARITY				PLIABILITY						HEIGHT			
0	1	2	3	0	1	2	3	0	1	2	3	4	5	0	1	2	3
Normal*	Pink	Red	Purple	Normal*	Pink	Red	Purple	Normal	Supple - flexible with minimal resistance	Yielding - giving way to pressure	Firm - inflexible, not easily moved, resistant to manual pressure	Banding - rope-like tissue that blanches with extension of scar	Contracture - permanent shortening of scar producing deformity or distortion	Normal - flat	<2 mm	<5 mm	>5 mm

Table S5. Scar Cosmesis Assessment and Rating Scale [12].

Clinical items	Scale ratings
Scar spread	0, None to near-invisible<br>1, Pencil-thin line<br>2, Mild spread, noticeable on close inspection<br>3, Moderate spread, obvious scarring<br>4, Severe spread
Erythema	0, absent<br>1, Light pink, some telangiectasias may be present<br>2, Red, many telangiectasias may be present<br>3, Deep red or purple

Dyspigmentation (includes hyperpigmentation and hypopigmentation)	0, Absent<br>1, Present
Track marks or suture marks	0, Absent<br>1, Present
Hypertrophy/Atrophy	0, Absent<br>1, Mild: palpable, barely visible hypertrophy or atrophy<br>2, Moderate: clearly visible hypertrophy or atrophy<br>3, Severe: marked hypertrophy or atrophy or keloid formation
Overall impression	0, Desirable scar<br>1, Undesirable scar
Patient items:	
Have you been bothered by any itch from the scar in the past 24 h?	0, No<br>1, Yes
Have you been bothered by any pain from the scar in the past 24 hours?	0, No<br>1, Yes

Table S6. Body Image Questionnaire [14].

**Body Image Questionnaire**			
**1- Have you felt less satisfied with your body since the surgery? (Do you think it is worse?)**			
1	2	3	4
no	yes, slightly worse	yes, moderately worse	yes, significantly worse
**2- Do you think the operation harmed your body?**			
1	2	3	4
no	yes, slightly	yes, moderately	yes, significantly
**3-Do you feel less attractive as a result of the surgery?**			
1	2	3	4
no	yes, slightly	yes, moderately	yes, significantly
**4-Do you feel less feminine as a result of the surgery?**			
1	2	3	4
no	yes, slightly	yes, moderately	yes, significantly
**5-Can you look at yourself naked, and do you feel bad when you look?**			
1	2	3	4
no	yes, slightly	yes, moderately	yes, significantly

Table S7. *Body-Cathexis Scale (15).*

	5=I strongly like it, don't like it	4=I moderately like it	3=I'm neutral,	2=I don't like it very much	1=I don't like it at all
1.My hair					
2. My face color					
3. My appetite					

4. My hands					
5. My body hair distribution					
6. My nose					
7. My physical strength					
8. My urinary and bowel habits					
9. My muscle strength					
10. My waist					
11. My energy level					
12. My back					
13. My ears					
14. My age					
15. My jaw					
16. My body structure					
17. My profile					
18. My height					
19. My sensory acuity					
20. My pain tolerance					
21. My shoulder width					
22. My arms					
23. My breasts					
24. My eye shape					
25. My digestive system					
26. My hips					
27. My disease resistance					
28. My legs					
29. My teeth shape					
30. My sexual potency					
31. My feet					
32. My sleep patterns					
33. My voice					
34. My health					
35. My sexual activity					

36. My knees					
37. My body posture					
38. My face shape					
39. My weight					
40. My genitals					

Table S8. Rosenberg Self-Esteem Scale [13]

ROSENBERG SELF-ESTEEM SCALE

1. I feel that I'm a person of worth, at least on an equal plane with others

a. Agree b. Strongly Agree c. Disagree d. Strongly Disagree

2. I feel that I have a number of good qualities.

a. Agree b. Strongly Agree c. Disagree d. Strongly Disagree

3. I am inclined to feel that I am a failure.

a. Agree b. Strongly Agree c. Disagree d. Strongly Disagree

4. I am able to do things as well as most other people.

a. Agree b. Strongly Agree c. Disagree d. Strongly Disagree

5. I do not have much to be proud of.

a. Agree b. Strongly Agree c. Disagree d. Strongly Disagree

6. I take a positive attitude toward myself.

a. Agree b. Strongly Agree c. Disagree d. Strongly Disagree

7. On the whole, I am satisfied with myself.

a. Agree b. Strongly Agree c. Disagree d. Strongly Disagree

8. I wish I had more respect for myself.

a. Agree b. Strongly Agree c. Disagree d. Strongly Disagree

9. I certainly feel useless at times.

a. Agree b. Strongly Agree c. Disagree d. Strongly Disagree

10. At times I think I am no good at all.

a. Agree b. Strongly Agree c. Disagree d. Strongly Disagree

Disclaimer/Publisher's Note: The statements, opinions and data contained in all publications are solely those of the individual author(s) and contributor(s) and not of MDPI and/or the editor(s). MDPI and/or the editor(s) disclaim responsibility for any injury to people or property resulting from any ideas, methods, instructions or products referred to in the content.
